# Research on Risk Transfer Pathways for Lung Cancer Among Middle-Aged and Older Individuals Using Deep Reinforcement Learning: Retrospective Cohort Study

**DOI:** 10.2196/74990

**Published:** 2026-04-15

**Authors:** Songjing Chen, Sizhu Wu

**Affiliations:** 1 Institute of Medical Information, Chinese Academy of Medical Sciences & Peking Union Medical College, No. 3 Yabao Road, Chaoyang District, Beijing, 100020, China, 86 01052328760

**Keywords:** lung cancer, risk transfer pathway, deep reinforcement learning, deep Q-learning, middle-aged and older individuals

## Abstract

**Background:**

Nowadays, lung cancer has a significantly high incidence rate worldwide. The mortality rate of lung cancer continues to rise; it is more common in middle-aged and older individuals and poses a great threat to human health.

**Objective:**

This study aimed to assess the lung cancer risk among middle-aged and older individuals in a timely manner and to establish an efficient pathway for the risk transfer.

**Methods:**

We proposed a deep reinforcement learning model based on deep Q-network (DQN) to explore the risk transfer pathway for lung cancer among middle-aged and older individuals. Risk stratification of lung cancer occurrence was deduced through deep neural network. The DQN model was developed using the Health and Retirement Study cohort for model training and internal validation. We also used the China Health and Retirement Longitudinal Study cohort for model external validation. Transfer simulation of multiple pathways in different cycles was calculated in a stratified risk groups–leveraged DQN model.

**Results:**

We developed and evaluated the DQN method for optimizing the risk transfer pathway among middle-aged and older individuals, with accuracy ranging from 0.917 (95% CI 0.896‐0.928) to 0.949 (95% CI 0.909‐0.961) and area under curve ranging from 0.906 (95% CI 0.887‐0.933) to 0.927 (95% CI 0.893‐0.938). External validation was conducted to assess the model’s effectiveness and availability. A total of 8780 and 3763 samples from the Health and Retirement Study were used for model training and internal testing, respectively, and 16,442 samples from the China Health and Retirement Longitudinal Study were used for external validation. The results showed that DQN models illuminated the optimal risk transfer pathways for stratified risk groups. Lung cancer incidence in the high risk group had declined by 68.2% through risk transfer of model-based simulation, which had declined by 56.9% in the medium risk group. Through simulative intervention and risk transition deduced from the DQN model, lung cancer incidences of the high risk, medium risk, and low risk groups were obviously decreased.

**Conclusions:**

A DQN-based deep reinforcement learning model was proposed and validated to develop and simulate a risk transfer pathway of lung cancer among middle-aged and older individuals. Risk stratification supplied an effective foundation for lung cancer risk transition.

## Introduction

Nowadays, lung cancer has a significantly high incidence rate worldwide. The mortality rate of lung cancer continues to rise; it is more common in middle-aged and older individuals and poses a great threat to human health. Moreover, 50% of patients with nonsmall cell lung cancer are aged 65 years and older at the time of initial diagnosis [1,2]. The pathogenesis of lung cancer is insidious, which is prone to misdiagnosis with nonspecific clinical manifestations. Therefore, it is important that we timely assess the risk for middle-aged and older individuals and establish an efficient pathway of the risk transfer for lung cancer.

Actively carrying out the risk assessment of lung cancer will help reduce the incidence rate of lung cancer. In the United Kingdom, a research team from the University of Oxford used the Cox proportional-hazards method in a population-based cohort to develop a lung cancer risk assessment model [3]. Henriksen et al [4] adopted a Bayesian network for lung cancer risk prediction based on a Danish population according to risk factors, such as age, sex, smoking, and laboratory results, and so on. Most previous studies conducted lung cancer risk assessment through traditional statistical methods or other machine learning methods [5-7] and lacked detailed stratification of lung cancer risk. A deep learning–based lung cancer risk assessment model with high performance was developed in this research, which provided a sufficiently detailed division for the risk process of lung cancer occurrence. Risk transfer in this research referred to lung cancer risk changes among high risk, medium risk, low risk, and non-risk states. High risk, medium risk, and low risk reflected that the risk of lung cancer decreased from high to low, and non-risk meant there was no risk of developing lung cancer. Deep Q-network (DQN) of deep reinforcement learning was adopted to explore risk transfer pathway to avoid lung cancer occurrence, which could effectively help reduce the incidence rate of lung cancer.

Research on the risk transfer pathways of lung cancer mainly focused on risk transitions among different risk states before lung cancer occurrence, as well as risk transitions between these risk states and lung cancer occurrence. However, several studies [8,9] were focused on the risk transition between high risk and lung cancer incidence. Huang et al [10] used lung cancer–associated risk factors, temporal trends, global incidence, and the mortality of lung cancer based on multiple open-access datasets to conduct research on high risk detection and analyze the trend of lung cancer incidence. Kowada [11] developed the state transition model to assess the cost-effectiveness of lung cancer screening in never smokers of the United States and Japan. Wang and Lukito [12] proposed the semi-Markov model to perform lung cancer risk and economic evaluation, which provided a health care decision support tool for the survivability and expenditure of lung cancer. Related research rarely explored the optimal risk transfer pathway through refined lung cancer risk to prevent disease occurrence [13-15]. Therefore, research on the risk transfer pathway for lung cancer among middle-aged and older individuals was conducive to reducing lung cancer incidence and helping early diagnosis and treatment of lung cancer.

The purpose of this research was to assess the lung cancer risk and develop a risk transfer pathway using deep reinforcement learning for middle-aged and older individuals. External validation was simulatively conducted to evaluate the DQN-based model through the large longitudinal cohort. We also explored risk transfer pathway evaluation to demonstrate effectiveness and availability of the proposed approach.

## Methods

### Data Sources

We used retrospective datasets from the Health and Retirement Study (HRS) [16] of the United States for model training and internal testing, which were selected between the year of 1992 and 2020 and followed up every 2 years, composing a population of more than 20,000. The cohort of the China Health and Retirement Longitudinal Study (CHARLS) [17] was adopted for model external validation, which involved a population of over 17,000 aged 45 years and older from 2010 to 2020. Lung cancer incidence, lung cancer survival, and overall survival were involved in this research. The inclusion criteria were aged 50 years and older and having respiratory disease–related questionnaire responses in follow-up surveys. Those without complete follow-up data were excluded. Risk factors such as person number, birth year, gender, marital status, BMI, years of education, smoking status, physical activity, drinking status, preventive behaviors, cancer history, and lung disease condition were involved in risk prediction and transfer modeling. We used R console to extract and map variables between the HRS and CHARLS datasets. For example, in HRS, the variable “PN” was used to represent a person number, while in CHARLS, the variable “ID” was used to denote a person number, as shown in Table 1. For years of education, specific education years were listed in HRS, while education types were supplied in CHARLS. We matched “no formal education illiterate” in CHARLS and “0 year of education” in HRS, and other education types were also matched similarly. Smoking cessation duration was calculated through “respondent smoking ever” and “respondent smoking now”; for example, if a respondent reported smoking ever in survey wave 1 and reported not smoking now in survey wave 2, we then deduced the respondent’s smoking cessation duration. Preventive behavior for cholesterol was involved in HRS and missed in CHARLS, which had no significant correlation with this research. Therefore, we did not include such variable.

**Table 1.** Variable comparison between HRS^a^ and CHARLS^b^.

Variables	Code in HRS	Code in CHARLS
Person number	PN	ID
Respondent’s reported birth year	RABYEAR	RABYEAR
Respondent’s reported gender	RAGENDER	RAGENDER
BMI in survey wave 1	R1BMI	R1MBMI
Marital status in survey wave 1	R1MSTAT	R1MSTAT
Years of education	RAEDYRS	RAEDUC_C
Respondent smoking ever in survey wave 1	R1SMOKEV	R1SMOKEV
Respondent smoking now in survey wave 2	R2SMOKEN	R2SMOKEN
Physical activity in survey wave 1	R1VGACTF	R1VGACT_C
Preventive behavior for cholesterol in survey wave 1	R1CHOLST	—^c^

a HRS: Health and Retirement Study.

b CHARLS: China Health and Retirement Longitudinal Study.

c Not applicable: the variable of “R1CHOLST” was involved in HRS, which was not applicable in CHARLS.

### Experimental Design

**Figure 1.**
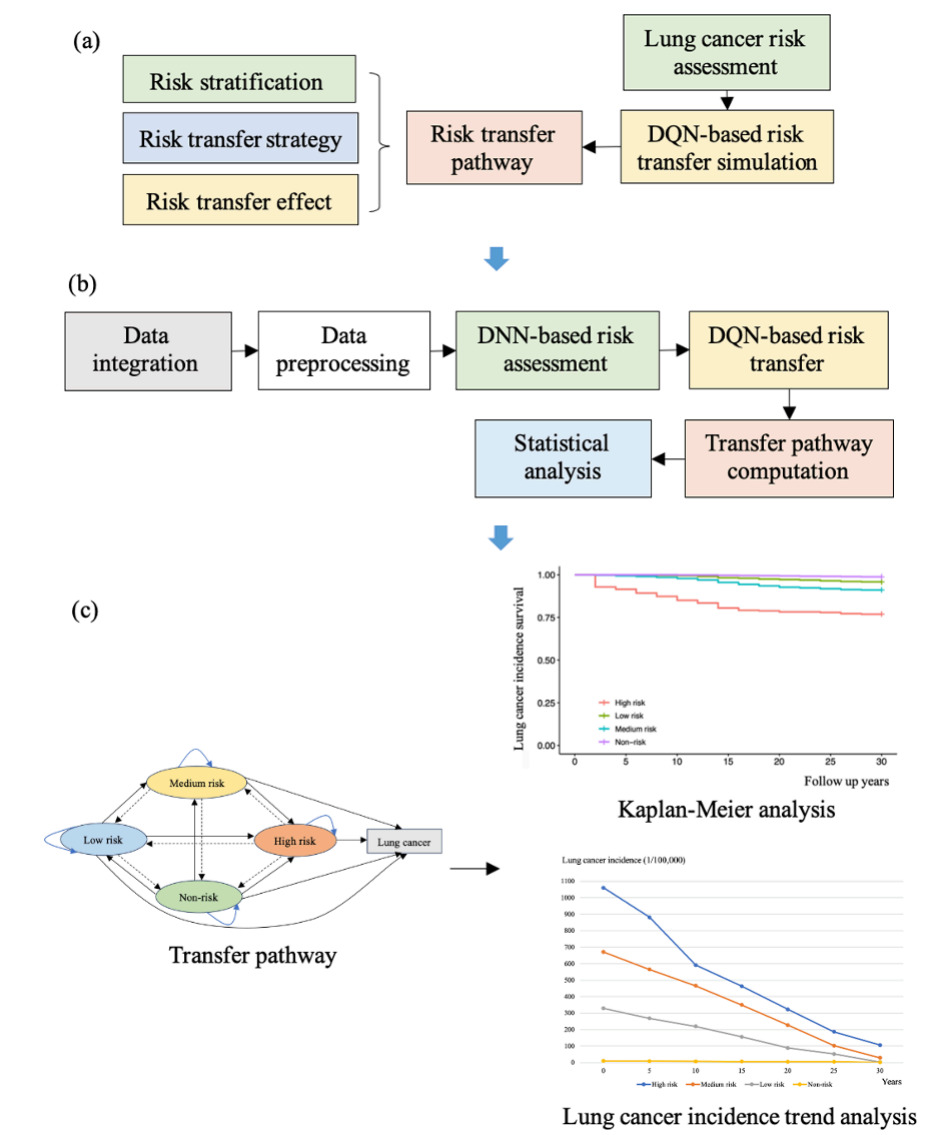
The main research technology route: (a) the outline of this research; (b) schematic diagram of the deep q-network model’s construction; and (c) survival analysis and lung cancer incidence trend analysis based on the risk transfer pathway. DNN: deep neural network; DQN: deep Q-network.

Lung cancer risk stratification was conducted based on the deep neural network (DNN) model and the risk transfer pathway was simulated using the DQN model. Lung cancer incidence, lung cancer survival, and overall survival were calculated to evaluate lung cancer risk stratification. Lung cancer incidence survival referred to the time from the initial follow-up to lung cancer diagnosis. Lung cancer survival indicated the interval between the initial follow-up and death from lung cancer or the last follow-up. Overall survival represented the time from the initial follow-up to death from any cause or the last follow-up. Four stratified risk degrees of lung cancer were generated using the weight thresholds of the DNN model, which included high risk, medium risk, low risk, and non-risk. Further, 3 risk transfer directions were deduced and analyzed through the DQN model, including direction of risk improvement, direction of risk deterioration, and risk maintenance (maintaining the original risk state). Risk improvement represented the risk decrease of lung cancer occurrence; risk deterioration reflected the risk increase of lung cancer occurrence; and risk maintenance indicated that the risk of lung cancer remained unchanged. The accuracy and the area under the receiver operating characteristic curve (AUROC) were computed to evaluate the performance of the DQN model. Risk transfer pathway for lung cancer was developed based on DQN models, as shown in Figure 1.

### Lung Cancer Risk Assessment

**Figure 2.**
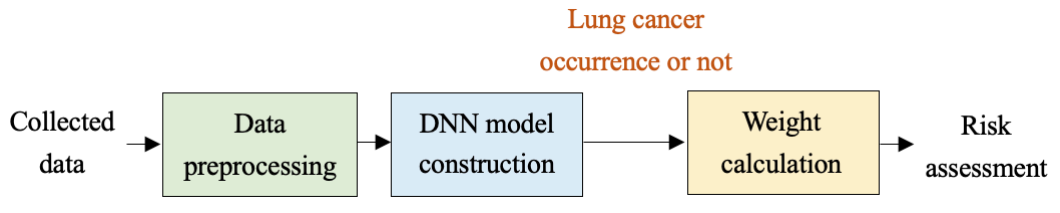
Schematic diagram of the lung cancer risk assessment model. DNN: deep neural network.

The risk assessment model of lung cancer occurrence was constructed based on the DNN method as shown in Figure 2. Because of the long follow-up time in HRS and CHARLS, it was inevitable that some indicators might have missing values. We adopted the multiple imputation method [18] to fill in the missing values. We used the mean value to perform initial imputation of missing values and used linear regression for iterative imputation. Then we updated the imputation value until realizing model convergence and reaching the maximum iteration. Therefore, missing values were predicted through multiple iterations. We also used singularly valuable decomposition [19] to reduce the white noises. We matched follow-up data according to each unique person number. Data imbalance problems were mitigated through the synthetic minority oversampling technique [20]. Datasets of HRS were randomly divided into the training set and the internal test set for the DNN-based risk assessment model, which included 8780 samples in the training set and 3763 samples in the internal test set. Further, 16,442 samples from CHARLS were adopted for external testing. The synthetic minority oversampling technique was used with the HRS training dataset to construct models. The k-nearest neighbor algorithm was applied to calculate the minority sample. The simulated samples were added to the HRS training dataset.

**Figure 3.**
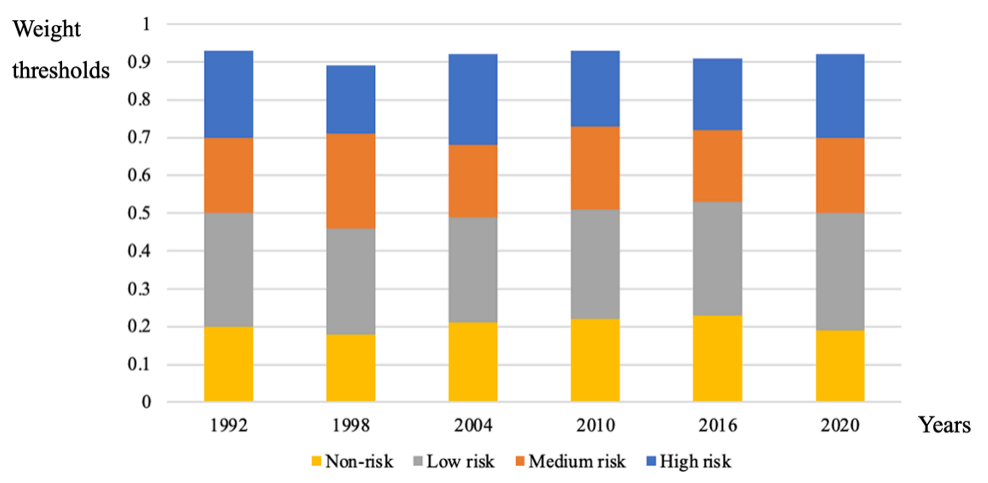
Weight thresholds of risk assessment in follow-up years.

The preprocessed data were input to the DNN model’s input layer. We iteratively calculate the weight matrix of risk factors using Equation (1). Specifically, *W_k_* and *b_k_* were the weight matrix and the bias vector of the *k*th layer of the DNN model. We used Equation (1) to compute the output of the *k*th layer. The linear rectification function, as shown in Equation (2), was used as the activation function to improve representation capability of the DNN model. Through multiple iterations and calculations, we computed weight thresholds using Equation (3), which are shown in Figure 3. When the average weight of the sample was in the interval 0‐0.2 in the year of 1992, we defined it as non-risk. When the average weight was in the interval 0.2‐0.5, 0.5‐0.71, or 0.71‐0.93 in the year of 1992, we defined it as low risk, medium risk, or high risk, respectively. Similarly, risk stratifications of other follow-up years were obtained according to weight thresholds as shown in Figure 3. The DNN-based lung cancer risk assessment model’s outputs were risk stratification of lung cancer occurrence, including high risk, medium risk, low risk, and non-risk. The calibration plots of the DNN model with Brier score and log loss are shown in Multimedia Appendix 1, and the confusion matrix of the DNN model is listed in Multimedia Appendix 2. The random seed of the DNN model was 42. Person number, birth year, gender, marital status, BMI, years of education, smoking status, physical activity, drinking status, prevention behaviors, cancer history, and lung disease condition, and so on, were involved features. The initial learning rate was 0.01, the batch size was 32, and the initial episode was 30. Through multiple iterations, 3 layers and 22 nodes were derived from the hidden layer, 56 nodes of the input layer, and 1 node of the output layer. The activation function was the linear rectification function. The *deepnet* package and h2o framework were leveraged for DNN model construction.


(1) $$l_{k}=W_{k}l_{k-1}+b_{k}$$

(2) $$f_{T}(x)=\left\{ \begin{array}{ll} 0, & x\leq 0\\ x, & x§gt;0 \end{array}\right.$$

(3) $$\begin{array}{rl} y & =f(x,\theta )\\ =f_{L-1}\left(W_{L-1}f_{L-2}\left(\cdots f_{1}\left(W_{1}X+b_{1}\right)\right)+b_{L-1}\right) \end{array}$$

### DQN Model Development and Validation

DQN models were constructed through different risk groups, and internal and external testing were conducted for model validation. Datasets including 8780 samples from HRS were used for DQN model training. A total of 3763 samples from HRS were used as an internal test set and 16,442 samples from CHARLS were used as an external test set. DQN models were built through 4 risk groups. First, Q-learning and convolutional neural networks (CNNs) were combined to construct the DQN model. The stratified risk group was the model’s input. We used CNN to approximate the target value function (target Q function) of the transfer strategy as closely as possible. Target Q functions for different risk groups were obtained. Second, the DQN model was trained using Q-learning. We calculated the loss function through Equation (4). *a* was the transfer strategy corresponding to different cycles. *s* was the different risk states including high risk, medium risk, low risk, and non-risk. *Q* was the output value function, which represented the maximum cumulative transfer effect of transfer strategy *a. Q(s,a; θ_i_*) was the output of the current network. *Q_i_* was the output of the target network. Third, we adopted the stochastic gradient descent function to optimize the loss function. The target *Q_i_* function of the transfer strategy was obtained using Equation (5), and the transfer strategies of different risk states were derived accordingly.

**Figure 4.**
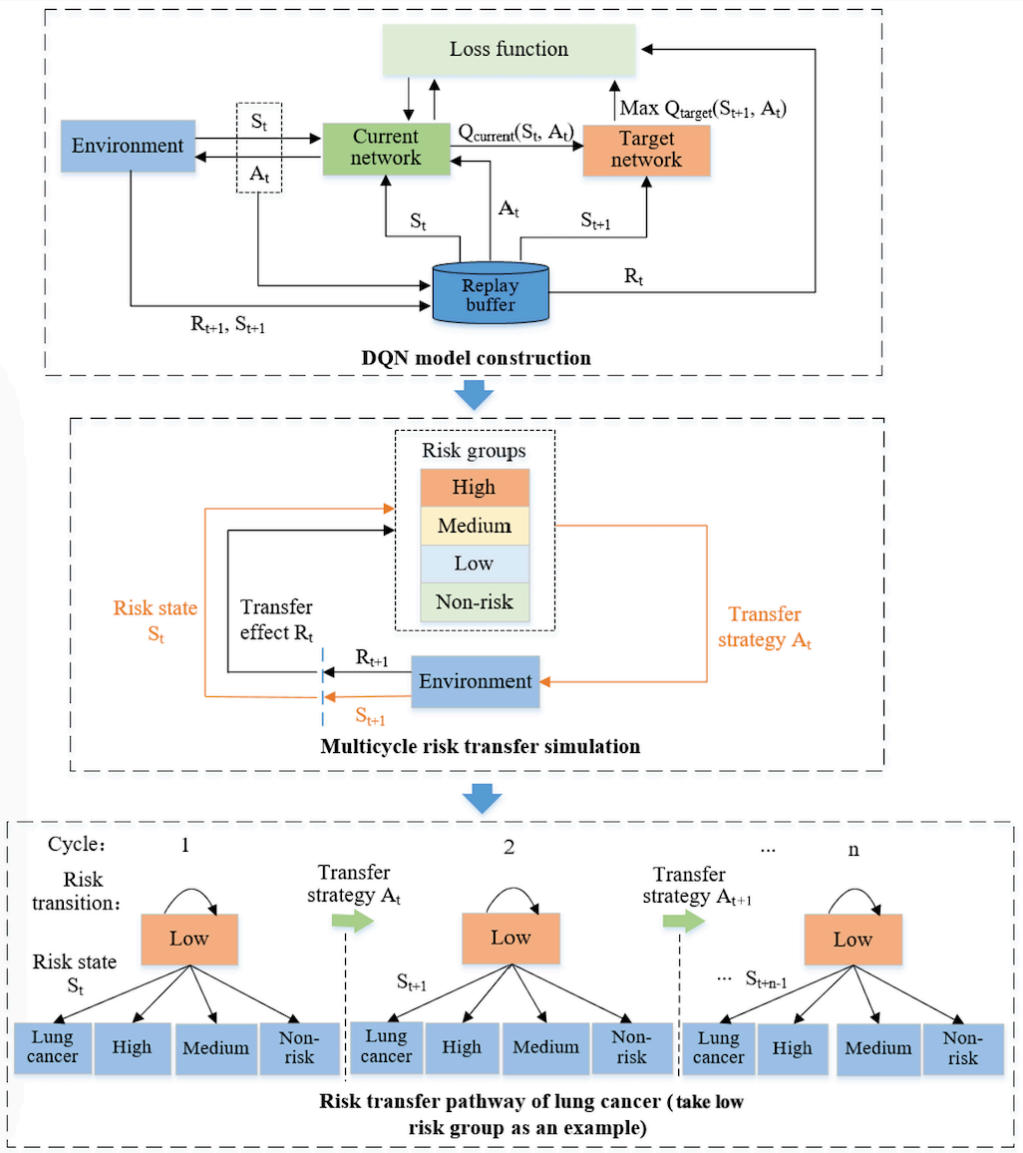
The workflow of deep Q-network model development and validation processes. DQN: deep Q-network.

The state space of the DQN setup was the vector (high risk state, medium risk state, low risk state, and non-risk state), and the action space was the set of transfer strategies, such as extending the duration of smoking cessation, reducing the frequency of smoking, and so on. We constructed DQN models for the 4 risk groups separately. As shown in Figure 4, we took the low risk group as an example to illustrate state representation and transition processes. We simulated risk transition of the low risk group in multiple cycles through corresponding transfer strategies. Similarly, DQN models for the high risk group, medium risk group, and non-risk group were developed to explore risk transfer pathways of lung cancer. The reward function is described in Equation (6), which was the transfer effect of current network. We used the stochastic gradient descent function to optimize the loss function. *Q(s’, a’; θ_i-1_*) was the output of the target network, *Q(s,a; θ_i_*) was the output of the current network, *r* was the transfer effect of the current network, *ε* was the transfer environment, and γ was the discount factor between 0.9 and 1. The random seed of the DQN model was 50. Person number, birth year, gender, marital status, BMI, years of education, smoking status, physical activity, drinking status, prevention behaviors, cancer history, and lung disease condition, and so on, were involved features. The initial learning rate was 0.01, the batch size was 256, and the episode number was 500. One input layer with 32×32 neurons; 3 convolutional layers with 5×5, 4×4, 3×3 convolution kernels; and 1 output layer were leveraged for the DQN model. OpenAI Gym and PyTorch framework were used.


(4) $$L_{i}\left(\theta_{i}\right)=E_{s,a∼\rho \left(\cdot \right)}\left[{\left(Q_{i}-Q\left(s,a;\theta_{i}\right)\right)}^{2}\right]$$

(5) $$Q_{i}=E_{s^{\prime }∼\varepsilon }\left[r+\gamma \underset{a^{\prime }}{max}Q\left(s^{\prime },a^{\prime };\theta_{i-1}\right)|s,a\right]$$

(6) $$\begin{array}{rl} \nabla_{\theta_{i}}L_{i}(\theta_{i}) & =E_{s,a∼\rho (\cdot );\;s^{\prime }∼\varepsilon }[\\ \;\left(r+\gamma \max_{a^{\prime }}Q(s^{\prime },a^{\prime };\theta_{i-1})-Q(s,a;\theta_{i})\right)\nabla_{\theta_{i}}Q(s,a;\theta_{i})] \end{array}$$

### Risk Transfer Pathways Computation

Transfer simulation of multiple pathways in different cycles for lung cancer occurrence was conducted in stratified risk groups through the DQN model. To explore optimal transfer strategy and transfer effect of different pathways, we calculated and deduced the optimum transfer pathway. The probabilities of lung cancer risk state transition for different risk groups were calculated for each transition cycle. Transfer pathways corresponding to various transfer directions, such as risk improvement, risk deterioration, and risk maintenance, were deduced. Then, the optimal transfer pathway and transfer effect were obtained through the DQN model.

### Statistical Analysis

Kaplan-Meier analysis was leveraged to demonstrate survival differences among the 4 stratified risk groups. We used a Cox proportional hazards model for the relative risk analysis between lung cancer incidence and smoking. R version 4.3.3 (R Core Team) was used for statistical analysis. The *deepnet* package and the h2o framework were used for DNN model training. OpenAI Gym and PyTorch framework were used for the DQN model. The *survival* and *survminer* packages were adopted for Kaplan-Meier analysis.

### Ethical Considerations

This study used secondary anonymized data from HRS and CHARLS, which were approved by the Institutional Review Board Committees of Michigan State University (HUM00061128) and the Biomedical Ethics Review Committee of Peking University (IRB00001052–11015). In this study, the individual data were anonymized; patients and the public were not involved in the design, conduct, and reporting stages of the research. All participants provided informed consent, and the study was conducted in accordance with the ethical principles outlined in the Declaration of Helsinki.

## Results

### Baseline Characteristics of Samples

Baseline characteristics of the training set, internal test set, and external test set are listed in Table 2. A total of 12,543 samples from HRS were used for DQN model development, and 16,442 samples from CHARLS were used for external testing of the DQN model.

**Table 2.** Baseline characteristics of the HRS^a^ training set, internal test set, and external test set.

Variables	HRS training set (n=8780)	HRS test set (n=3763)	CHARLS^b^ test set (n=16,442)
Age group (years), n (%)			
≤45	369 (4.20)	158 (4.19)	1090 (6.63)
45‐65	8141 (92.72)	3489 (92.72)	11,431 (69.52)
>65	270 (3.08)	115 (3.06)	3766 (22.9)
Missing	1 (0.01)	—^c^	155 (0.94)
Sex, n (%)			
Male	4069 (46.34)	1744 (46.35)	7884 (47.95)
Female	4711 (53.66)	2019 (53.65)	8556 (52.04)
Missing	—	—	2 (0.01)
Ethnicity, n (%)			
Non-Hispanic White	6976 (82.12)	2990 (83.46)	—^d^
Black	1456 (14.37)	624 (13.27)	—
Other	348 (3.51)	149 (3.27)	—
Region, n (%)			
Northeast	1497 (17.05)	641 (17.03)	—
Midwest	2194 (24.99)	940 (24.98)	—
South	3662 (41.71)	1570 (41.72)	—
West	1427 (16.25)	612 (16.27)	—
Missing	—	—	—
Habitation, n (%)			
Urban	—	—	7171 (43.61)
Rural	—	—	9271 (56.39)
Marital status, n (%)			
Married	6815 (77.62)	2921 (77.62)	12,966 (78.86)
Married, spouse absent	40 (0.46)	17 (0.45)	—
Partnered	264 (3.01)	113 (3)	1243 (7.56)
Separated	217 (2.47)	93 (2.47)	71 (0.43)
Divorced	756 (8.61)	324 (8.61)	143 (0.87)
Widows	435 (4.95)	186 (4.94)	1856 (11.29)
Never married	253 (2.88)	109 (2.90)	153 (0.93)
Missing	—	—	10 (0.06)
Years of education, n (%)			
0‐3	169 (1.92)	73 (1.94)	6578 (40)
4‐6	385 (4.38)	165 (4.38)	4657 (28.32)
7‐9	894 (10.18)	383 (10.18)	3119 (18.97)
10‐12	3637 (41.42)	1559 (41.43)	1266 (7.7)
13‐16	2848 (32.44)	1220 (32.42)	794 (4.83)
≥17	820 (9.34)	351 (9.33)	12 (0.07)
Missing	28 (0.32)	12 (0.32)	16 (0.10)

a HRS: Health and Retirement Study.

b CHARLS: China Health and Retirement Longitudinal Study.

c Not available.

d Not applicable: the “ethnicity” for HRS in Table 2 consisted of “White,” “Black,” and “other,” which were not applicable for CHARLS.

### Lung Cancer Risk Stratification

**Figure 5.**
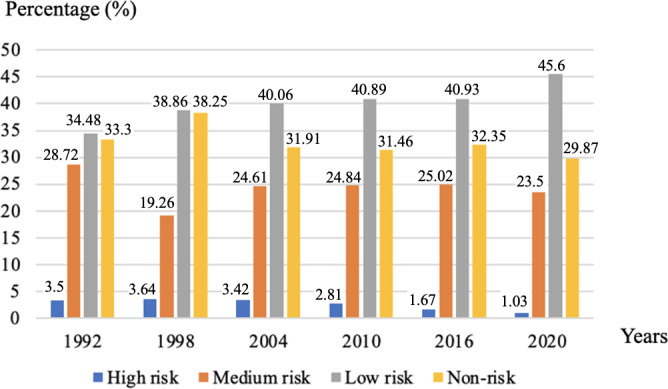
The percentage of risk transfer for lung cancer incidence in different risk groups.

**Figure 6.**
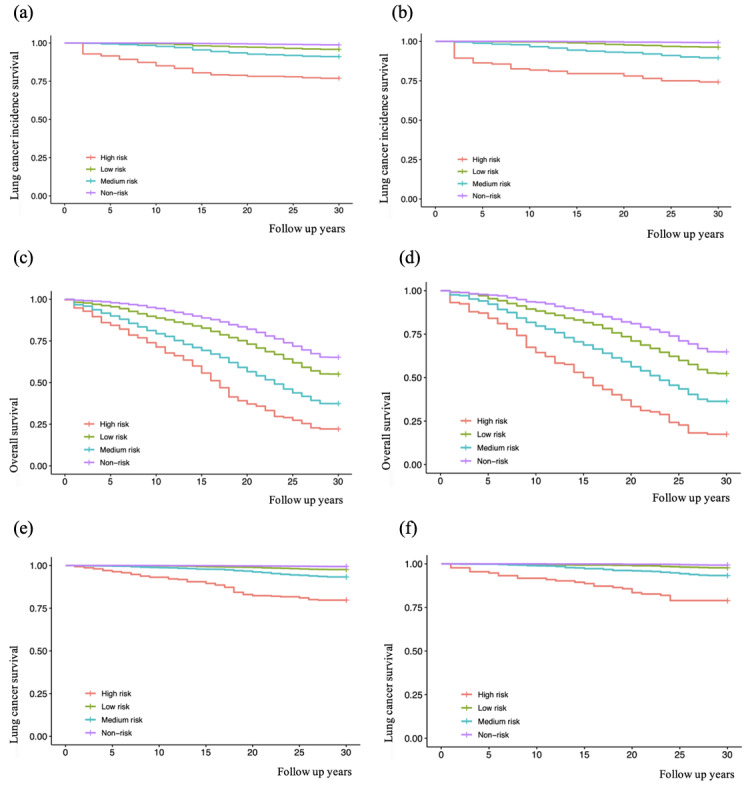
K-M analysis of the high risk, medium risk, low risk, and non-risk subgroups of the HRS cohort: (a) K-M curve of lung cancer incidence survival in HRS training set; (b) K-M curve of lung cancer incidence survival in the HRS test set; (c) K-M curve of overall survival in the HRS training set; (d) K-M curve of overall survival in the HRS test set; (e) K-M curve of lung cancer survival in the HRS training set; and (f) K-M curve of lung cancer survival in the HRS test set. HRS: Health and Retirement Study; K-M: Kaplan-Meier.

The risk stratification and the trend of risk transfer of lung cancer incidence in different follow-up years are shown in Figure 5, which displays the percentages of the high risk, medium risk, low risk, and non-risk groups. People in the high risk group had higher lung cancer incidence than the medium risk, low risk, and non-risk groups, as shown in Figure 6A and B. The horizontal axis represents the follow-up time, and the value 1.00 on the vertical axis indicates the absence of lung cancer. The value 0.75 represents that the incidence survival rate of lung cancer was 75%, and the corresponding incidence rate of lung cancer was 25%. Lung cancer incidence of the medium risk group was also higher than low risk and non-risk groups. At the last follow-up, lung cancer incidence of the high risk group was about 25%, which was much higher than the incidence of the low risk and non-risk groups. As shown in Figure 6C and D, the high risk group had lower overall survival than other risk groups. Moreover, lung cancer survival of the high risk group was also lower than medium risk, low risk, and non-risk groups, as shown in Figure 6E and F.

**Figure 7.**
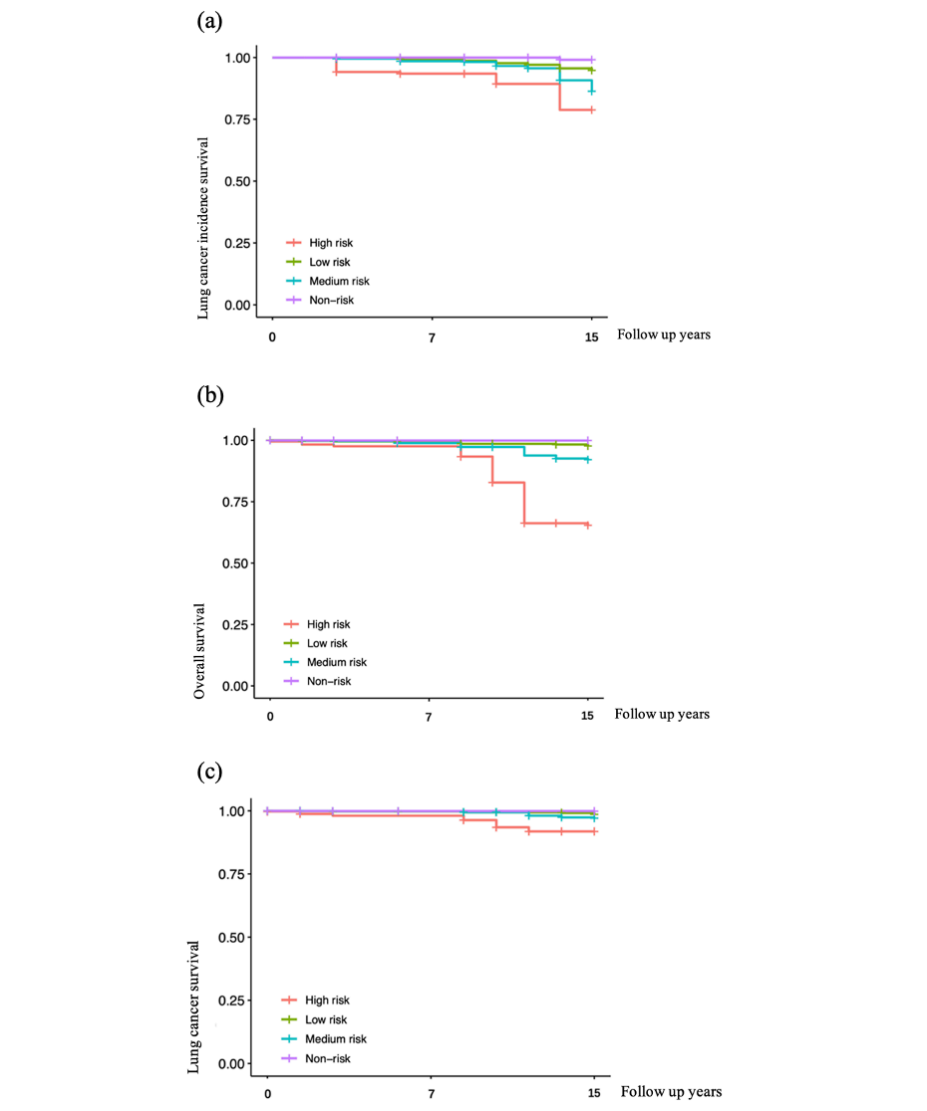
K-M analysis of the high risk, medium risk, low risk, and non-risk subgroups in the external validation CHARLS cohort: (a) K-M curve of lung cancer incidence survival in the external validation CHARLS cohort; (b) K-M curve of overall survival in the external validation CHARLS cohort; and (c) K-M curve of lung cancer survival in the external validation CHARLS cohort. CHARLS: China Health and Retirement Longitudinal Study; K-M: Kaplan-Meier.

Furthermore, external testing of the DNN model was conducted using the CHARLS cohort, as shown in Figure 7. External test results and internal test results of lung cancer risk assessment based on DNN models were mainly consistent.

### Risk Transfer Strategy and Pathway

**Figure 8.**
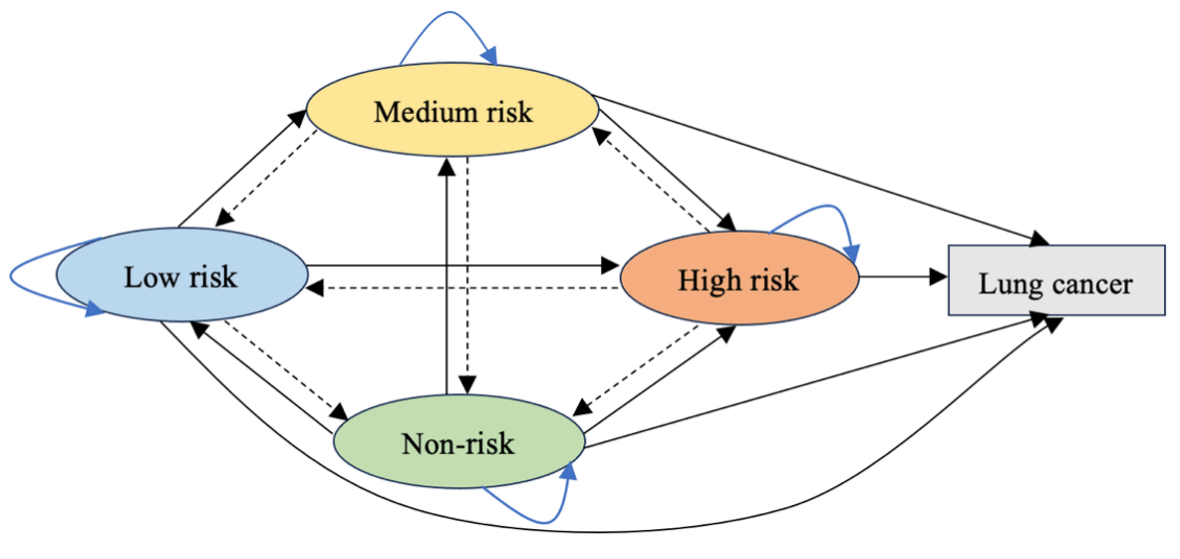
Risk transfer pathways of lung cancer incidence. Black dashed arrows represent risk improvement, black solid arrows represent risk deterioration, and blue solid arrows represent risk maintenance.

Lung cancer risk transfer strategy and pathway were explored through DQN models. We deduced different transfer pathways of lung cancer incidence for stratified risk groups, as shown in Figure 8. Risk improvement represented the reduced risk of lung cancer occurrence, including high risk to medium risk, then to low risk and non-risk; high risk to low risk, then to non-risk; high risk to non-risk; medium risk to low risk, then to non-risk; medium risk to non-risk; and low risk to non-risk. Risk deterioration reflected the increased risk of lung cancer occurrence, including non-risk to low risk, then to medium risk and high risk; non-risk to medium risk, then to high risk; non-risk to high risk; low risk to medium risk, then to high risk; low risk to high risk; medium risk to high risk; high risk to lung cancer occurrence; medium risk to lung cancer occurrence; low risk to lung cancer occurrence; and non-risk to lung cancer occurrence. Risk maintenance represented that the risk of lung cancer occurrence had not changed, including high risk, medium risk, low risk, and non-risk remaining unchanged separately. In the risk improvement pathways, extending the duration of smoking cessation—such as quitting smoking from less than 1 month to 1 year, quitting smoking from less than 1 month to 3 years, or quitting smoking from less than 1 month to more than 5 years—and reducing the frequency of smoking—such as from everyday smoking to someday smoking, from someday smoking to quitting smoking, or from everyday smoking to quitting smoking—were the effective intervention strategies.

**Figure 9.**
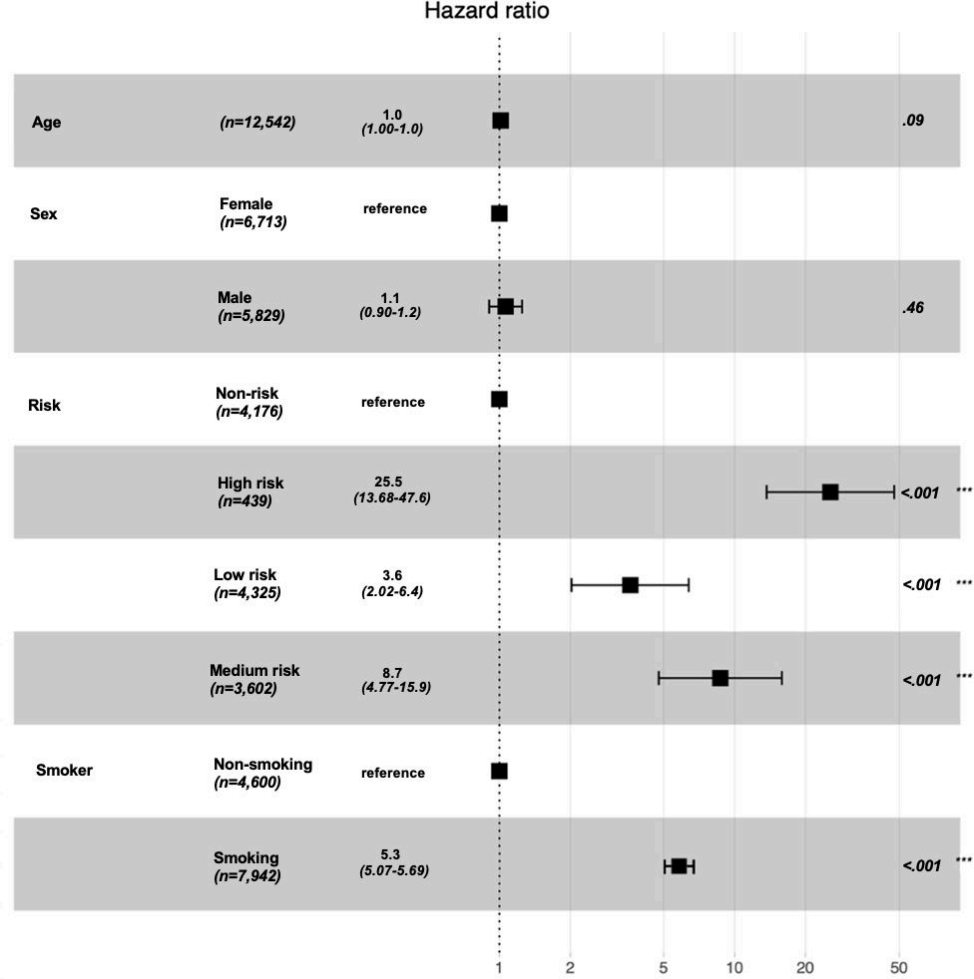
Forest plot of lung cancer incidence and smoking.

The accuracy and AUROC of DQN models are shown in Multimedia Appendix 3. We used the decrease of lung cancer incidence to evaluate the effectiveness of the transfer strategy. The transfer strategy brought the largest decline of lung cancer incidence compared to others, which could be considered an optimal transfer strategy. Otherwise, the transfer strategy should be adjusted through a feedback mechanism. The whole workflow would be reworked as shown in Figure 4; the transfer pathway was comprehensively evaluated until an optimal transfer strategy was derived. For off-policy evaluation, DQN used experience replay data to update the strategy, and existing historical data were reused, as shown in Figure 4. Extending the duration of smoking cessation and reducing the frequency of smoking were the optimal transfer strategies for risk improvement pathway. The tangible intervention strategies were extending the duration of smoking cessation from less than 1 month to more than 5 years and reducing smoking frequency from everyday smoking to quitting. A forest plot of lung cancer incidence and smoking is shown in Figure 9. Lung cancer incidence of the high risk group had the closest relationship with smoking than the medium risk, low risk, and non-risk groups. The optimal transfer pathways and the decline of lung cancer incidence of stratified risk groups are displayed in Table 3. Lung cancer incidence of the high risk group had declined from 1060.38 per 100,000 to 336.78 per 100,000, which had decreased by 68.2% through risk transfer. The lung cancer incidence of the medium risk group had reduced from 671.20 per 100,000 to 289.13 per 100,000, which had declined by 56.9%.

**Table 3.** Optimal transfer pathway and transfer effect of stratified risk groups.

Transfer pathway	Lung cancer incidence after transfer (per 100,000)	Incidence decline (%)
High risk→medium risk→low risk→non-risk	336.78	68.24
Medium risk→low risk→non-risk	289.13	56.92
Low risk→non-risk	3.02	99.08

### Transfer Pathway Evaluation

**Figure 10.**
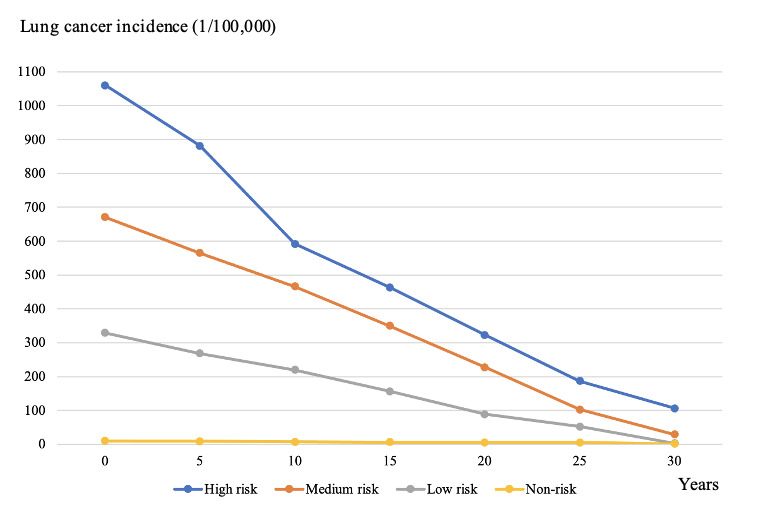
The incidence trend of lung cancer for stratified risk groups.

The incidence trend of lung cancer for stratified risk groups was simulated and analyzed to evaluate the optimal transfer pathway and transfer strategy, as shown in Figure 10. According to the optimal transfer strategy and the optimal transfer pathway, lung cancer incidence of the high risk group reduced to 106.78 per 100,000 after simulative intervention and risk transition, lung cancer incidence of the medium risk group declined to 29.13 per 100,000, and the incidence of the low risk group also decreased obviously. The results validated the effectiveness and availability of the transfer pathway.

### Comparison With Other Methods

We used random forest to conduct comparison work of the DQN-based risk transfer pathway simulation. The training dataset and internal test dataset of HRS were leveraged to construct a random forest model for lung cancer incidence, lung cancer survival, and overall survival. The CHARLS dataset was used for external validation. Risk transfer pathways of lung cancer incidence were derived through random forest models, and optimal transfer pathways were also deduced, which were mainly the same as those of the DQN models. For example, in the risk improvement pathway, extending the duration of smoking cessation and reducing the frequency of smoking were the optimal transfer strategies through random forest models, which were consistent with the DQN models. The accuracies of random forest external validation models for stratified risk groups were from 0.823 to 0.851, and the AUROCs were from 0.778 to 0.836, which were almost lower by 10% than the DQN models, as shown in Table 4. Therefore, DQN models had better performance for transfer pathways computation than random forest models.

**Table 4.** Comparison of performance between random forest models and deep Q network models of external validation.

Risk groups	Random forest		DQN^b^	
	Accuracy (95% CI)	AUROC^a^ (95% CI)	Accuracy (95% CI)	AUROC (95% CI)
High risk	0.837 (0.791‐0.852)	0.793 (0.776‐0.826)	0.949 (0.909‐0.961)	0.927 (0.893‐0.938)
Medium risk	0.823 (0.803‐0.865)	0.812 (0.785‐0.845)	0.936 (0.913‐0.952)	0.919 (0.886‐0.941)
Low risk	0.826 (0.796‐0.848)	0.778 (0.761‐0.827)	0.917 (0.896‐0.928)	0.913 (0.902‐0.937)
Non-risk	0.851 (0.825‐0.876)	0.836 (0.793‐0.859)	0.921 (0.902‐0.946)	0.906 (0.887‐0.933)

a AUROC: area under the receiver operating characteristic curve.

b DQN: deep Q network.

## Discussion

Lung cancer risk transfer pathways were simulated through proposed DQN models for stratified risk groups among middle-aged and older individuals. The DQN-based risk transfer pathway calculation model performed well, not only for internal validation of the HRS dataset but also for external validation of the CHARLS dataset, with accuracy ranging from 0.917 (95% CI 0.896‐0.928) to 0.949 (95% CI 0.909‐0.961) and AUROC ranging from 0.906 (95% CI 0.887‐0.933) to 0.927 (95% CI 0.893‐0.938). Moreover, optimal risk transfer pathways through model-based simulations were effective solutions to reducing lung cancer incidence for the high risk, medium risk, low risk, and non-risk groups. The optimal intervention strategies for decreasing lung cancer incidence were extending the duration of smoking cessation from less than 1 month to more than 5 years and reducing smoking frequency from everyday smoking to quitting, which could have significant effects in the high risk and medium risk groups.

We developed DNN models for lung cancer risk stratification, which refined different lung cancer risk factors and supplied research foundation for lung cancer risk transition. The optimal risk transfer strategy and the optimal transfer effect were deduced using DQN models, which improve the effectiveness and availability of the proposed risk transfer pathway. In recent years, deep learning has been adopted by more research [21-24] for lung cancer risk prediction. Landy et al [25] used the recalibrated CNNs to identify risk individuals in the National Lung Screening Trial and predict lung cancer risk. Bhatia et al [26] developed and validated lung cancer risk screening and prediction model using transfer learning. Jiang et al [27] applied CNNs to predict lung cancer risk, and the National Lung Screening Trial cohort was used to evaluate the model’s performance. The DQN model, which we proposed to explore risk transfer pathway, could also demonstrate the stratified risk classification and the optimal transfer strategy for lung cancer prevention.

We developed and evaluated the DQN model for optimizing the risk transfer pathway among middle-aged and older individuals. External validation was conducted to assess the model’s effectiveness and availability. The results illuminated that DQN models could deduce the optimal risk transfer pathways for stratified risk groups. Lung cancer incidence of the high risk group had declined by 68.2% through risk transfer, which had declined by 56.9% in the medium risk group. Lung cancer incidences of high risk, medium risk, and low risk were obviously decreased through simulated risk intervention and transition, which were consistent with previous studies [14,28,29] and indicated that actively quitting smoking before being diagnosed with lung cancer could effectively reduce the incidence and mortality of lung cancer.

Previous studies adopted statistical models or other machine learning models to predict lung cancer risk, such as multiple logistic regression [30-32], artificial neural network [33], random forest [34,35], extreme gradient boosting [36,37], decision tree [38], and risk prediction models [39]. Compared to these models, the proposed DQN model explored risk transfer pathways of lung cancer among middle-aged and older individuals with high accuracy and AUROC. External validation was conducted to verify the proposed model based on stratified risk states, which enhanced the credibility and effectiveness of the DQN model. Moreover, proposed risk transfer pathways might supply clues for revealing the pathogenesis of lung cancer.

Several limitations existed in this research. First, this study explored lung cancer risk pathways of middle-aged and older individuals based on a large longitudinal retrospective cohort, which did not involve a causal framework. A causal framework, such as directed acyclic graphs, could be constructed in the further research. Second, variables available in the HRS and CHARLS survey datasets were mostly demographic and self-reported health questionnaire data in this study. Genomic mutation data, chest computed tomography scan image, outpatient attendance records, laboratory tests, and so on, could be involved in future work to improve the biological plausibility and real-world use of the derived risk transfer pathways. Third, conducting follow-up surveys over the years for the large longitudinal cohort was a long-term process. Transfer pathway evaluation was conducted through simulative prediction at present. In the future, this evaluation could be done according to a real-world longitudinal cohort. Fourth, lung cancer incidence had a significant decrease through the proposed optimal risk transfer pathway, which needs clinical validation in our future work.

In conclusion, a DQN-based deep reinforcement learning model was proposed and validated to develop and simulate the risk transfer pathway of lung cancer among middle-aged and older individuals. Risk stratification supplied an effective foundation for lung cancer risk transition. This modeling approach of the risk transfer pathway might supply a prevention clue for other chronic diseases.

## Supplementary material

10.2196/74990 Multimedia Appendix 1 Calibration plots of the deep neural network model.

10.2196/74990 Multimedia Appendix 2 Confusion matrix of the deep neural network model.

10.2196/74990 Multimedia Appendix 3 Deep Q-network models’ performance for internal and external validation.

## Abbreviations

AUROC: area under the receiver operating characteristic curve
CHARLS: China Health and Retirement Longitudinal Study
CNN: convolutional neural network
DNN: deep neural network
DQN: deep Q-network
HRS: Health and Retirement Study
