# Supplementary figures and images for: Research on Risk Transfer Pathways for Lung Cancer Among Middle-Aged and Older Individuals Using Deep Reinforcement Learning: Retrospective Cohort Study
Source: JMIR Med Inform. 2026 Apr 15;14:e74990. doi: 10.2196/74990 (PMC13082448; doi:10.2196/74990)

**Figure S1.** Calibration plots of DNN model


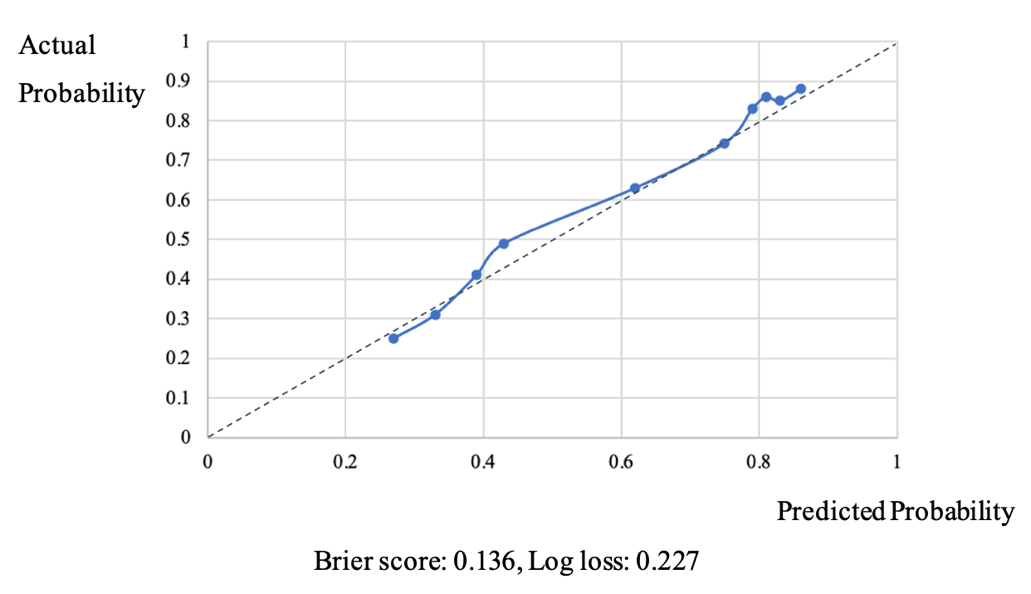

Supplement: Multimedia Appendix 1 [file medinform-v14-e74990-s001.docx]
