# Supplementary material for: Research on Risk Transfer Pathways for Lung Cancer Among Middle-Aged and Older Individuals Using Deep Reinforcement Learning: Retrospective Cohort Study
Source: JMIR Med Inform. 2026 Apr 15;14:e74990. doi: 10.2196/74990 (PMC13082448; doi:10.2196/74990)
Supplement: Multimedia Appendix 2 [file medinform-v14-e74990-s002.docx]

**Table S1.** Confusion matrix of DNN model

| Confusion matrix | Predicted lung cancer | Predicted without lung cancer |
| --- | --- | --- |
| Actual lung cancer | 356 | 41 |
| Actual without lung cancer | 47 | 15998 |
