# Supplementary material for: Research on Risk Transfer Pathways for Lung Cancer Among Middle-Aged and Older Individuals Using Deep Reinforcement Learning: Retrospective Cohort Study
Source: JMIR Med Inform. 2026 Apr 15;14:e74990. doi: 10.2196/74990 (PMC13082448; doi:10.2196/74990)
Supplement: Multimedia Appendix 3 [file medinform-v14-e74990-s003.docx]

Table S1. DQN models performance of internal validation and external validation.

| Risk groups | Internal validation | | External validation | |
| --- | --- | --- | --- | --- |
|  | Accuracy (95% CI) | AUROC^a^ (95% CI) | Accuracy (95% CI) | AUROC (95% CI) |
| High risk | 0.946(0.912-0.958) | 0.923(0.891-0.947) | 0.949(0.909-0.961) | 0.927(0.893-0.938) |
| Medium risk | 0.937(0.904-0.959) | 0.915(0.903-0.935) | 0.936(0.913-0.952) | 0.919(0.886-0.941) |
| Low risk | 0.922(0.903-0.947) | 0.921(0.895-0.939) | 0.917(0.896-0.928) | 0.913(0.902-0.937) |
| Non-risk | 0.923(0.893-0.942) | 0.917(0.891-0.938) | 0.921(0.902-0.946) | 0.906(0.887-0.933) |

^a^AUROC: area under the receiver operating characteristic curve.

*P*<.001 was considered to indicate statistical significance.
